# TRIM59 promotes immune evasion and tumor progression in lung adenocarcinoma via ubiquitin- proteasomal degradation of IRF3

**DOI:** 10.3389/fimmu.2026.1813280

**Published:** 2026-05-20

**Authors:** Xue Li, Meifang Guo, Zhen Lian

**Affiliations:** 1 Department of Radiation Oncology, Tianjin’s Clinical Research Center for Cancer, Key Laboratory of Cancer Prevention and Therapy, Tianjin Medical University Cancer Institute and Hospital, National Clinical Research Center for Cancer, Tianjin, China; 2 Department of Ultrasonic Diagnosis, The Second People’s Hospital of Dongying, Dongying, Shandong, China; 3 Department of Emergency, Tianjin’s Clinical Research Center for Cancer, Key Laboratory of Cancer Prevention and Therapy, Tianjin Medical University Cancer Institute and Hospital, National Clinical Research Center for Cancer, Tianjin, China

**Keywords:** immune evasion, IRF3, lung adenocarcinoma, prognostic biomarker, single-cell RNA sequencing, TRIM59, tumor microenvironment, ubiquitination

## Abstract

Lung adenocarcinoma (LUAD) remains a leading cause of cancer-related mortality, with immune evasion being a key driver of treatment resistance and poor prognosis. The tripartite motif-containing (TRIM) family of E3 ubiquitin ligases plays critical roles in regulating tumor immunity, but the functional relevance and molecular mechanisms of most TRIM members in LUAD remain elusive. Here, we integrated multi-omics analyses with functional experiments to systematically investigate the prognostic value and immunoregulatory role of TRIM family in LUAD. We identified TRIM59 as an independent poor prognostic factor, with its high expression correlating with an immunosuppressive tumor microenvironment (TME). Mechanistically, TRIM59 interacts with interferon regulatory factor 3 (IRF3) and promotes its ubiquitination and proteasomal degradation, thereby inhibiting the IRF3-STING pathway and downstream anti-tumor interferon production. Single-cell analyses revealed cell-type-specific functions of IRF3: in tumor cells, IRF3 may suppresses immune-activating gene expression and modulates proliferation; in tumor-infiltrating T/NK cells, IRF3 negatively regulates pro-inflammatory signaling. Collectively, our findings establish the TRIM59-IRF3 axis as a novel regulatory pathway driving LUAD immune evasion, providing a promising prognostic biomarker and therapeutic target for enhancing immunotherapy efficacy.

## 1 Introduction

Lung adenocarcinoma (LUAD), the most prevalent subtype of non-small-cell lung cancer (NSCLC), accounts for approximately 40% of all lung cancer cases worldwide (1). This disease continues to pose a significant healthcare challenge, with a 5-year survival rate of less than 50% (2–4). Despite significant advancements in immunotherapies such as immune checkpoint inhibitors (ICIs), only a subset of LUAD patients derive durable clinical benefits, primarily due to the complex mechanisms of tumor immune evasion (5). Therefore, dissecting the molecular networks that regulate the tumor microenvironment (TME) is crucial for identifying novel prognostic biomarkers and therapeutic targets to improve patient outcomes.

Ubiquitination, a reversible post-translational modification orchestrated by E1 activating enzymes, E2 conjugating enzymes, and E3 ligases, plays a pivotal role in regulating tumor immunity (6–8). It modulates key immune processes including immune cell activation, antigen presentation, immune checkpoint dynamics, and innate immune signaling pathways such as the cyclic GMP-AMP (cGAMP) synthetase (cGAS) - stimulator of interferon genes (STING) axis (9–11). The tripartite motif (TRIM) family proteins, characterized by a conserved RING finger domain, B-box domain, and coiled-coil domain, constitutes a large group of E3 ubiquitin ligases with diverse functions in cancer progression and immune regulation (12). For instance, TRIM56 has been shown to promote Src activation via K63-linked ubiquitination to drive tumor progression in hepatocellular carcinoma (13); TRIM27 acts as a oncogenic driver in ovarian cancer through inactivating the cGAS/STING signaling pathway (14). However, the expression patterns, prognostic significance, and molecular targets of most TRIM family members in LUAD remain incompletely characterized.

Interferon regulatory factor 3 (IRF3), a key transcription factor downstream of the STING pathway, plays a central role in initiating anti-tumor immunity by inducing type I interferons (IFNs) and pro-inflammatory cytokines (15, 16). IRF3 activation triggers the recruitment and activation of immune effector cells, thereby enhancing anti-tumor immune surveillance. Dysregulation of IRF3 signaling, including impaired activation or increased degradation, has been implicated in immune suppression across multiple cancers (17, 18). However, the mechanism underlying IRF3 regulation in LUAD and its impact on the TME remain elusive.

In this study, by integrating multi-omics data from The Cancer Genome Atlas (TCGA), Gene Expression Omnibus (GEO), and Gene Set Cancer Analysis (GSCA) databases with functional experiments (co-immunoprecipitation, ubiquitination assays, Western blot) and single-cell analyses, we have analyzed the prognostic value and immunoregulatory mechanisms of TRIM family proteins in LUAD. Our results identify TRIM59 as an independent poor prognostic factor in LUAD and demonstrate that it promotes IRF3 ubiquitination and degradation, thereby inhibiting the STING-IRF3 pathway and driving immune evasion. Furthermore, single-cell analyses reveal context-dependent functions of IRF3 in tumor cells and tumor-infiltrating immune cells, highlighting the complexity of the TRIM59-IRF3 regulatory axis. These findings provide a novel mechanistic basis for LUAD immune evasion and offer a potential therapeutic target for enhancing immunotherapy efficacy.

## 2 Materials and methods

### 2.1 Data collection and processing

The human TRIM gene family was retrieved from the HUGO Gene Nomenclature Committee (HGNC) database (https://www.genenames.org/), and the complete list of its members is provided in **Supplementary Table 1**. Gene expression data (RNA-seq) and corresponding clinical information for 541 LUAD tissue samples and 59 adjacent normal lung tissue samples were downloaded from TCGA Genomic Data Commons (GDC) Data Portal (https://portal.gdc.cancer.gov/). The GSCA database (http://bioinfo.life.hust.edu.cn/GSCA/), an integrated platform for genomic mutational landscape analysis, was used to assess the copy number variation (CNV) and single nucleotide variation (SNV) of TRIMs family. Single-cell RNA sequencing (scRNA-seq) data from 9 LUAD tumor specimens (9 patients) were retrieved from the GSE189357 dataset in the GEO database (https://www.ncbi.nlm.nih.gov/geo/) (19).

### 2.2 Single-cell RNA sequencing data analysis

scRNA-seq data were processed using the Seurat package (v4.0) in R (version 3.6.3). Briefly, UMI matrices were read to construct Seurat objects, followed by quality control (QC) to retain cells with <15% mitochondrial gene content and 200–7000 expressed genes. Batch effects were mitigated using the Harmony package (v0.1.0). Cell clusters were identified using the FindNeighbors and FindClusters functions, and visualized by UMAP embedding. Gene set activity at the single-cell level was quantified using Seurat’s AddModuleScore function. Differentially expressed genes (DEGs) between cohorts were identified using the FindMarkers function with the Wilcoxon test, and genes with adjusted p < 0.05 and |log2(fold change)| > 1 were considered significant.

### 2.3 Screening of prognostic TRIM family genes

A multistep strategy was adopted to identify prognostic TRIM family genes. Firstly, differential expression of TRIM family genes between LUAD tumor and adjacent normal samples was analyzed using the limma R package (v3.46.0) with TCGA-LUAD data. Genes with adjusted p < 0.05 and FoldChange > 2 were considered significant. Secondly, univariate Cox proportional hazards regression analysis was performed to assess the association between gene expression and patient survival risk. Samples were stratified into high- and low-expression groups based on the median expression value, and Kaplan-Meier (KM) survival curves were generated with log-rank test for significance. Genes were retained if they met: (1) p < 0.05 in both univariate Cox and KM survival analyses; (2) logHR (from Cox model) × logFC (from differential expression analysis) > 0 (consistent trend between expression alteration and survival risk). Lastly, multivariate Cox proportional hazards regression analysis was performed to verify the independent prognostic value of candidate genes, adjusting for clinical covariates (age, gender, tumor stage).

### 2.4 Immune infiltration analysis

The infiltration levels of 24 immune cell types were evaluated using ImmuCellAI (20). Spearman correlation analyses were performed to assess the associations between TRIM6, TRIM15, TRIM59 expression and immune cell infiltration, as well as immune-related gene signatures.

### 2.5 Single-cell functional analysis

Cell clusters from scRNA-seq data were annotated based on canonical marker genes: B cells (MS4A1, CD79B), myeloid cells (LYZ, CD14), T/NK cells (NKG7, CD3D), plasma cells (IGHG1, IGKC), endothelial (PECAM1, VWF), epithelial/tumor cells (KRT19, EPCAM), fibroblasts (COL1A2, DCN), and mast cells (TPSAB1, CPA3). The scTenifoldKnk package (21) was used to perform in silico IRF3 knockout experiments in purified tumor cells (annotated by LUAD-specific markers: NKX2-1, NAPSA, CEACAM5) and T/NK cells. DEGs were subjected to Gene Ontology (GO) and Kyoto Encyclopedia of Genes and Genomes (KEGG) pathway enrichment analyses using the clusterProfiler package.

### 2.6 Cell culture and plasmid transfection

Human NSCLC cell lines (PC9 and A549) were purchased from the American Type Culture Collection (ATCC) and authenticated by short tandem repeat (STR) profiling. PC9 cells were cultured in DMEM (Gibco, USA) supplemented with 10% fetal bovine serum (FBS, Gibco), and A549 cells were maintained in RPMI 1640 (Gibco) with 10% FBS. All cells were cultured at 37 °C in a humidified incubator with 5% CO_2_ and confirmed to be mycoplasma-free. The pCMV-TRIM59×Myc-SV40-Neo plasmid was purchased from GeneChem (Shanghai, China). Cells were seeded 24 h prior to transfection with Lipofectamine 3000 (Thermo Fisher Scientific) according to the manufacturer’s protocol.

### 2.7 Western blot analysis

Whole-cell lysates were extracted using SDS lysis buffer (Beyotime, China) supplemented with a complete protease inhibitor cocktail and phosphatase inhibitor (Roche, Switzerland). Protein concentrations were determined using the BCA Protein Assay Kit (Beyotime). Equal amounts of protein were separated by SDS-PAGE, transferred to PVDF membranes (Millipore, USA), blocked with 5% non-fat milk for 1 h at room temperature, and incubated with primary antibodies overnight at 4 °C. Membranes were then incubated with horseradish peroxidase (HRP)-conjugated secondary antibodies for 1 h at room temperature, and proteins were visualized using enhanced chemiluminescence (Millipore) on a Tanon 5200 chemiluminescent imaging system (Tanon, China). Primary antibodies used: rabbit anti-STING (D2P2F, 13647), rabbit anti-phospho-STING (Ser366, E9A9K, 50907), rabbit anti-IRF3 (D6I4C, 11904), rabbit anti-phospho-IRF3 (Ser396, D6O1M, 29047), rabbit anti-GAPDH (14C10, 2118) (Cell Signaling Technology, USA); rabbit anti-TRIM59 (28575-1-AP), rabbit anti-Myc (16286-1-AP) (Proteintech, USA); mouse anti-ubiquitin (P4D1, sc-8017), normal mouse IgG (sc-2025) (Santa Cruz Biotechnology, USA). MG132 (S2619) and 3-Methyladenine (3-MA, S2767) were purchased from Selleck (USA).

### 2.8 Co-Immunoprecipitation assay

Total protein was extracted from transfected cells using mild cell lysis buffer for Western and IP (Beyotime) supplemented with protease inhibitors. Protein extracts were incubated with indicated primary antibodies overnight at 4 °C in a rotator, captured by Protein A/G PLUS-Agarose (Santa Cruz Biotechnology, Cat# sc-2003), and subjected to SDS-PAGE followed by immunoblotting as described above.

### 2.9 Immunofluorescence staining

Cells were washed with ice-cold PBS, fixed with 4% paraformaldehyde for 10 min, permeabilized with 0.2% Triton X-100 for 10 min, and blocked with 1% BSA in PBST (PBS + 0.1% Tween 20) for 1 h. Cells were incubated with primary antibodies in 1% BSA in PBST overnight at 4 °C, followed by fluorescent secondary antibodies for 1 h at room temperature. Coverslips were mounted with ProLong™ Gold Antifade Mountant with DAPI (Thermo Fisher Scientific, Cat# P36931), and images were acquired with a Zeiss LSM710 confocal microscope (Zeiss, Germany).

### 2.10 Quantitative reverse transcription PCR

Total RNA was extracted using TRIzol Reagent (Thermo Fisher Scientific, Cat# 15596018) and reverse-transcribed into cDNA using PrimeScript™ RT Master Mix (Takara, Cat# RR036A). qRT-PCR was performed with TB Green^®^ Premix Ex Taq™ II (Takara, Cat# RR820A) on a Bio-Rad T100 thermal cycler (Bio-Rad, USA). mRNA expression was normalized to GAPDH using the 2^-^ΔΔCt method. Primers used are listed in **Supplementary Table 2**.

### 2.11 Molecular docking

Amino acid sequences of human IRF3 (Protein ID: AAH71721) and TRIM59 (Protein ID: NP_775107) were retrieved from the National Center for Biotechnology Information (NCBI). Protein-protein interactions were predicted using AlphaFold3 (22). The predicted structures were subsequently analyzed with PyMOL (v2.3.4) to identify the amino acid pairs involved in hydrogen bond formation between the interacting proteins (23). Additionally, the predicted multimeric structures were subjected to analysis using PRODIGY for the calculation of the binding energy of the protein-protein complex.

### 2.12 Statistical analysis

Statistical analyses were performed using R software (v3.6.3) and GraphPad Prism (v8.0). Differences between groups were analyzed using Student’s t-test, ANOVA, or Wilcoxon rank-sum test as appropriate. KM survival curves were generated with log-rank test for significance. Univariate and multivariate Cox proportional hazards regression analyses were used to assess prognostic value. Spearman’s correlation was used to evaluate associations between gene expression, immune cell infiltration, and immune-related signatures. “*”, “**”, and “***” indicate p < 0.05, < 0.01, and < 0.001, respectively. All statistical tests were two-tailed, and p < 0.05 was considered statistically significant.

## 3 Results

### 3.1 Expression and mutational profile of TRIM family genes in LUAD

To characterize the proteogenomic landscape of TRIM family genes in LUAD, we first analyzed their expression, CNV, and SNV profiles using TCGA and GSCA datasets. Differential expression analysis revealed that most TRIM family genes exhibited altered expression in LUAD tumors compared to adjacent normal tissues: TRIM58 and MEFV were significantly downregulated (p < 0.001), while TRIM2, TRIM6, TRIM9, TRIM15, TRIM17, TRIM31, TRIM45, TRIM46, TRIM47, TRIM54, and TRIM59 were upregulated (**Supplementary Figure 1A**, **Supplementary Table 3**). CNV analysis showed that TRIM11 had the highest heterozygous amplification ratio (61.6%), while TRIM13 had the highest heterozygous deletion ratio (53.1%) (**Supplementary Figure 2**, **Supplementary Table 4**). SNV analysis revealed a generally low mutation frequency (0–6%) across TRIM family genes, with CMYA5 and TRIM51 exhibiting the highest mutation frequency and TRIM49D1, TRIM51HP, and TRIM73 the lowest (**Supplementary Figure 1B**, **Supplementary Table 5**).

### 3.2 TRIM6, TRIM15, and TRIM59 are independent poor prognostic factor in LUAD

Our differential expression analysis identified 13 significantly dysregulated TRIM family genes. Univariate Cox regression and KM survival analyses further revealed that TRIM6, TRIM15, and TRIM59 were poor prognostic factors, with high expression correlating with significantly shorter overall survival (OS) (**Supplementary Figure 1C**, **Supplementary Table 6**). Multivariate Cox regression analysis, adjusting for age, gender, and tumor stage, confirmed that TRIM6 (HR = 1.35, 95% CI: 1.15–1.59, p < 0.001), TRIM15 (HR = 1.19, 95% CI: 1.05–1.36, p = 0.007), and TRIM59 (HR = 1.50, 95% CI: 1.17–1.92, p = 0.001) were independent prognostic factors (**Supplementary Table 7**).

### 3.3 TRIM59 modulates the dual facets of anti-tumor immunity in LUAD

**Figure 1:**
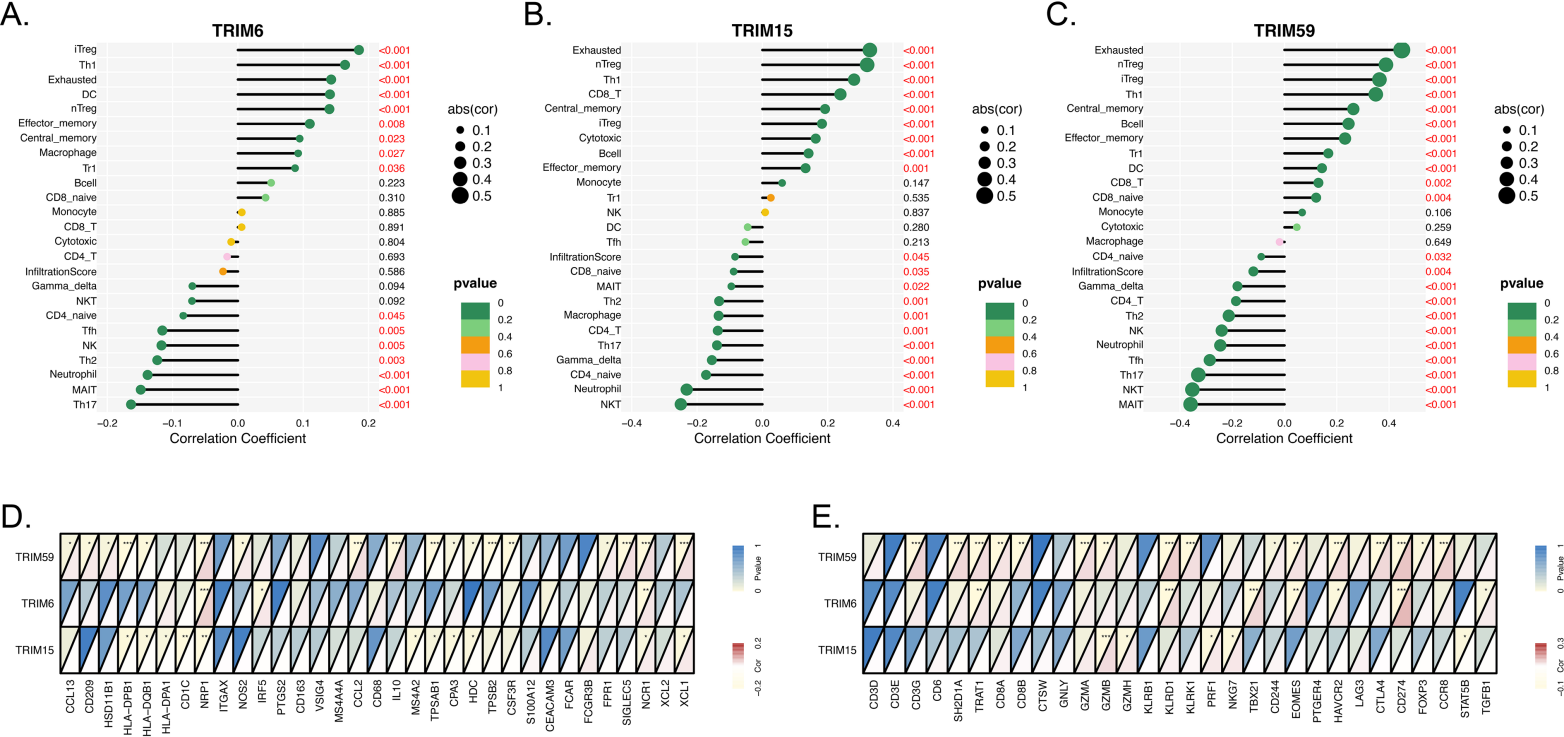
TRIM family members correlate with immune cell infiltration and immune-related gene expression in LUAD. **(A-C)** Correlation of TRIM6, TRIM15, and TRIM59 expression with immune cell infiltration in LUAD. The horizontal axis represents the Spearman correlation coefficient, with positive values indicating a positive correlation and negative values indicating a negative correlation. The size of each dot reflects the absolute value of the correlation coefficient [abs(cor)], and the color indicates the p-value, with green representing a significant correlation (p < 0.05). All correlations with p < 0.001 are labeled as such. **(D-E)** Correlation heatmaps of TRIM6, TRIM15, and TRIM59 expression with immune-related genes. The upper triangle displays the p-value (blue indicates significance), and the lower triangle displays the Spearman correlation coefficient (red indicates a positive correlation, blue indicates a negative correlation). “*”, “**”, and “***” indicate p < 0.05, < 0.01, and < 0.001, respectively.

Ubiquitination serves as a pivotal regulator of tumor immunity, governing multiple key processes including immune cell activation, antigen presentation, and immune checkpoint dynamics. To explore the potential roles of TRIM6, TRIM15, and TRIM59 within the tumor immune microenvironment of LUAD, we systematically interrogated their associations with immune cell infiltration patterns and immune-related gene expression profiles. Our analyses demonstrate that all three TRIM family members are significantly correlated with immune cell infiltration and immune gene signatures, yet TRIM59 consistently exhibits the most pronounced effects. In terms of immune cell infiltration (**Figures 1A–C**), TRIM59 displays the strongest positive correlations with exhausted T cells, naive regulatory T cells, and induced regulatory T cells, with correlation magnitudes that surpass those of TRIM15 and TRIM6. Conversely, TRIM59 also shows the most robust negative correlations, particularly with MAIT cells, NKT cells, and Th17 cells, whereas TRIM15 correlates negatively with NKT cells and neutrophils, and TRIM6 shows the weakest negative associations with Th17 and MAIT cells. When examining immune-related gene associations (**Figures 1D, E**), TRIM59 uniquely correlates with both T-cell activation markers and inhibitory exhaustion markers, indicating a dual role in modulating T-cell function and exhaustion. In contrast, TRIM15 is primarily linked to anti-inflammatory and chemotactic genes like IL10 and CCL2, while TRIM6 associates mainly with antigen-presentation genes such as HLA-DPB1 and HLA-DQA1. Collectively, these findings highlight TRIM59 as a central regulator in shaping the tumor immune microenvironment of LUAD, suggesting it may represent a promising therapeutic target for modulating anti-tumor immunity.

### 3.4 TRIM59 interacts with IRF3

**Figure 2:**
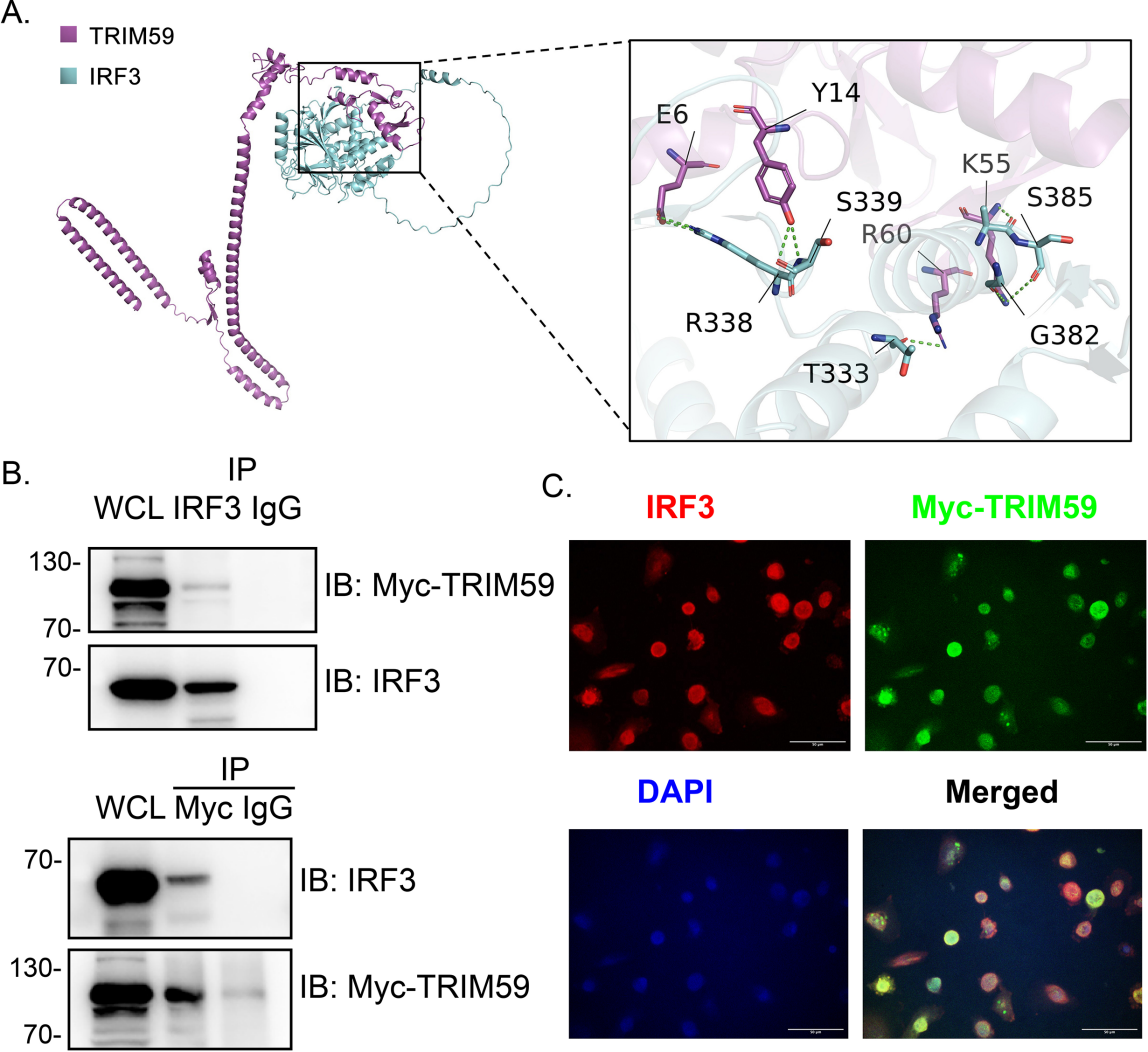
TRIM59 Interacts with IRF3. The interaction between TRIM59 and IRF3 was characterized using molecular docking, co-immunoprecipitation, and confocal microscopy. **(A)** Molecular docking model of the TRIM59-IRF3 complex (TRIM59: purple; IRF3: cyan), with an expanded view of the binding interface highlighting key interacting residues (TRIM59: E6, Y14, K55, R60; IRF3: R338, S339, T333, S385, G382). **(B)** Co-immunoprecipitation (coIP) assays confirm the physical interaction between TRIM59 and IRF3. **(C)** Confocal immunofluorescence microscopy shows co-localization of IRF3 (red) and Myc-TRIM59 (green) in cells, with DAPI (blue) staining nuclei. The merged image (right) demonstrates overlapping signals (yellow/orange), confirming their subcellular co-localization.

As an E3 ubiquitin ligase, TRIM59 exerts its biological functions primarily by catalyzing the ubiquitination of target substrates. To identify the potential downstream molecules of TRIM59, we employed the UbiBrowser database to predict candidate substrates that may interact with TRIM59 (**Supplementary Table 8**). Among these predicted substrates, IRF3 exhibited the highest scores in both Domain_Likelihood Ratio and Go_Likelihood Ratio analyses; thus, we further evaluated the binding potential between TRIM59 and IRF3 using molecular docking simulations. The results revealed a total of 60 intermolecular contact sites, with apolar-apolar contacts (19.0) as the dominant interaction type, indicating hydrophobic interaction served as the core driving force for the complex formation. The physicochemical property analysis of interaction residues (NIS) at the binding interface showed that apolar residues accounted for 42.47% and charged residues for 26.16%, which was consistent with the dominant hydrophobic interaction characteristics of the contact sites. For the binding capacity, the complex exhibited a high predicted binding affinity of -9.2 kcal·mol^-1^, and the predicted dissociation constant (Kd) was 1.7×10^-7^ M at 25.0 °C, demonstrating a stable and high-affinity binding between TRIM59 and IRF3 (**Figure 2A**). Additionally, coIP assays verified the physical interaction between TRIM59 and IRF3 (**Figure 2B**), while confocal microscopy confirmed their co-localization within cells (**Figure 2C**). Taken together, these lines of evidence conclusively demonstrate that TRIM59 interacts with IRF3.

### 3.5 TRIM59 attenuate IRF3 mediated antitumor immunity through ubiquitylation

**Figure 3:**
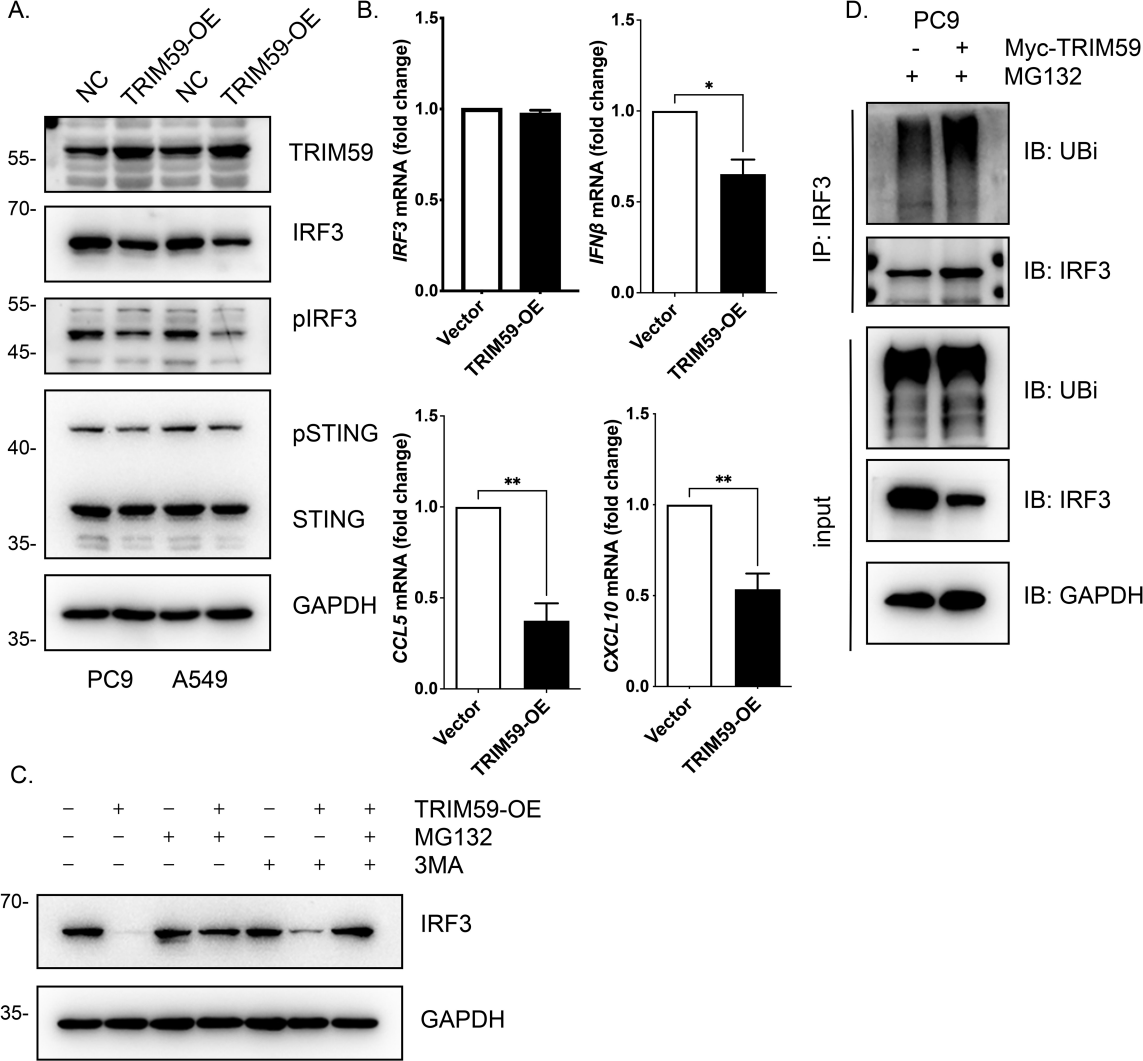
TRIM59 promotes ubiquitin-proteasome-mediated degradation of IRF3 to suppress the STING-IRF3 signaling pathway. **(A)** Immunoblot analysis of TRIM59 and STING-IRF3 signaling in PC9 and A549 cells transfected with either empty vector (NC) or TRIM59-overexpression (TRIM59-OE) constructs. **(B)** qRT-PCR analysis showing that TRIM59 overexpression had no effect on IRF3 mRNA levels, but significantly suppressed the transcription of downstream immune response genes IFNB, CCL5, and CXCL10 in PC9 cells. **(C)** Immunoblot analysis of IRF3 protein levels in TRIM59-OE cells treated with the proteasome inhibitor MG132 or the autophagy inhibitor 3-MA. MG132, but not 3-MA, rescued IRF3 from TRIM59-induced degradation, indicating a proteasome-dependent mechanism. **(D)** IP assay demonstrating that TRIM59 overexpression enhances IRF3 ubiquitination. PC9 cells transfected with Myc-TRIM59 and treated with MG132 were subjected to IP with an IRF3 antibody, followed by immunoblotting with an anti-ubiquitin (UBi) antibody. The results show increased polyubiquitination of IRF3 in the presence of TRIM59. “*”, “**”, and “***” indicate p < 0.05, < 0.01, and < 0.001, respectively.

To further clarify the impact of TRIM59 on IRF3 protein levels, we modulated TRIM59 expression *in vitro* and evaluated IRF3 abundance at both the protein and mRNA levels. Our results showed that ectopic overexpression of TRIM59 led to a significant reduction in IRF3 protein levels (**Figure 3A**), whereas it exerted no detectable effect on IRF3 mRNA expression (**Figure 3B**). In addition, TRIM59 overexpression attenuated IRF3-mediated activation of the STING signaling pathway and suppressed the production of downstream IFN cytokines (**Figures 3A, B**). These findings suggested that TRIM59 regulates the STING pathway by modulating IRF3 protein stability. Further mechanistic investigations revealed that treatment with the proteasome inhibitor MG132, but not the autophagy inhibitor 3-MA, completely abrogated TRIM59-induced IRF3 degradation (**Figure 3C**). Moreover, co-expression of TRIM59 markedly enhanced the ubiquitylation of IRF3 (**Figure 3D**). Collectively, these results demonstrated that TRIM59 promotes the ubiquitin-proteasome system (UPS)-mediated degradation of IRF3. In summary, our data conclusively indicate that TRIM59 suppresses the IRF3-STING pathway-mediated immune response by facilitating the ubiquitination and subsequent degradation of IRF3.

### 3.6 Single-cell analysis reveals cell-type-specific expression of IRF3

**Figure 4:**
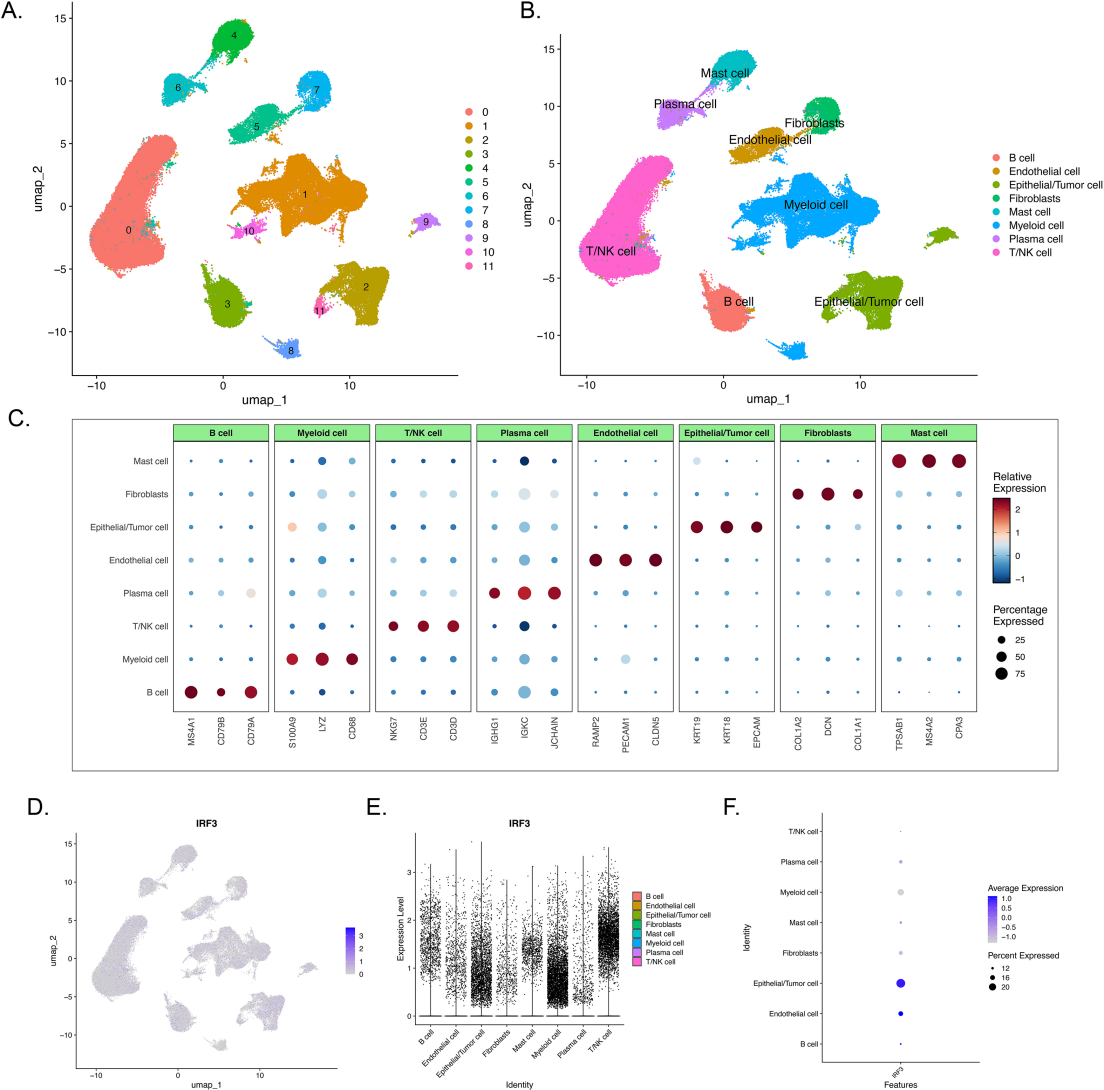
Single-cell RNA sequencing reveals the cellular landscape of lung adenocarcinoma and cell-type-specific expression of IRF3. **(A)** UMAP visualization of unsupervised clustering, identifying 12 distinct cell clusters. **(B)** UMAP plot displaying the annotated major cell types, including T/NK cells, myeloid cells, B cells, plasma cells, mast cells, fibroblasts, epithelial/tumor cells, and endothelial cells. **(C)** Dot plot showing the expression specificity of canonical marker genes across all annotated cell types, where dot size indicates the percentage of cells expressing the gene and color represents the relative expression level. **(D)** UMAP plot visualizing the expression distribution of IRF3 across all cells, with color intensity indicating expression level. **(E)** Violin plot demonstrating IRF3 expression levels across different cell types, revealing highest expression in immune cell subsets (B cells, T/NK cells, myeloid cells) and minimal expression in epithelial/tumor cells and fibroblasts. **(F)** Dot plot summarizing IRF3 expression, showing its enrichment in immune effector cells, with high average expression and detection percentage in B cells and T/NK cells.

To dissect the cellular landscape of LUAD across the full spectrum of malignant progression, we analyzed scRNA-seq data from the GSE189357 dataset, which captures the dynamic evolution of LUAD from adenocarcinoma *in situ* (AIS) and minimally invasive adenocarcinoma (MIA) to invasive adenocarcinoma (IAC). We included all samples along this progression continuum to characterize both early and persistent molecular events in tumorigenesis. In total, we analyzed 9 surgical specimens from 9 LUAD patients, yielding 107,370 high-quality cells. Unsupervised clustering identified 12 distinct cell clusters (**Figure 4A**), which were annotated into 8 major cell types using canonical lineage markers: B cells (MS4A1, CD79B), myeloid cells (LYZ, CD14), T/NK cells (NKG7, CD3D), plasma cells (IGHG1, IGKC), endothelial cells (PECAM1, VWF), epithelial/tumor cells (KRT19, EPCAM), fibroblasts (COL1A2, DCN), and mast cells (TPSAB1, CPA3) (**Figure 4B**). The dot plot confirms cell type specificity of marker genes, showing high expression of lineage-defining transcripts and minimal cross-cluster contamination (**Figure 4C**). To address potential concerns regarding biological heterogeneity introduced by including pre-invasive lesions, we performed a sensitivity analysis restricted to MIA and IAC samples. This analysis confirmed that the cellular composition and IRF3 expression patterns remained consistent regardless of AIS inclusion (**Supplementary Figure 3**), demonstrating that our core findings are robust and specific to invasive tumors.

IRF3 expression profiling revealed heterogeneous expression across cell subsets. While detectable in most cell populations, expression levels and the proportion of positive cells were relatively low across the dataset (**Figures 4D–F**). Notably, IRF3 exhibited the highest relative expression in immune effector cells, including B cells, T/NK cells, and myeloid cells, whereas expression was minimal to undetectable in epithelial/tumor cells and fibroblasts. This cell-type-specific expression pattern suggests that IRF3 may participate in regulating immune cell functions within the tumor microenvironment.

### 3.7 IRF3: immune regulation and proliferation-associated signatures in tumor cells

**Figure 5:**
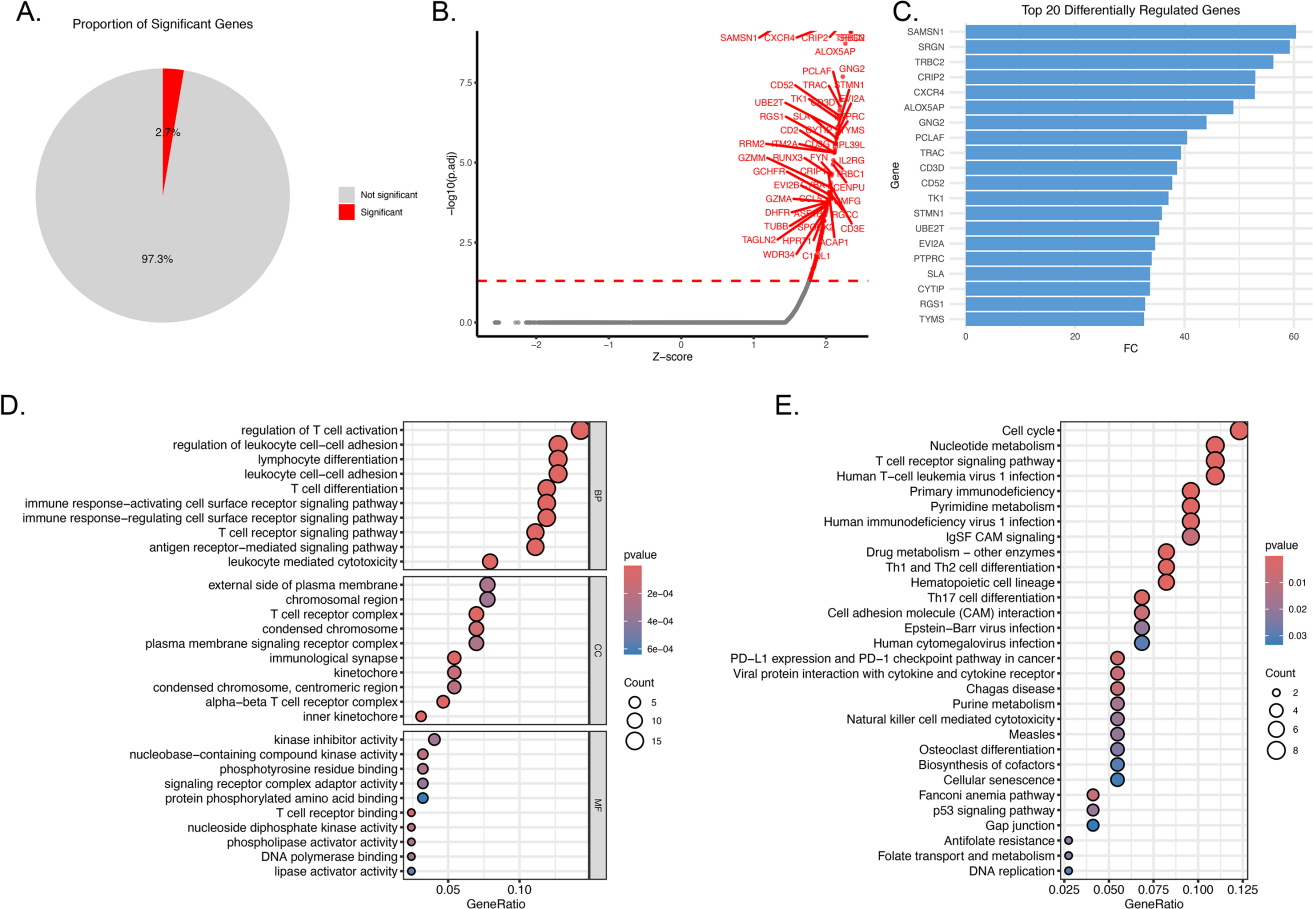
Virtual knockout of IRF3 reveals its role in suppressing immune-activating signaling and modulating tumor cell proliferation in LUAD. **(A)** Pie chart showing that IRF3 depletion altered the expression of 2.7% of genes, with the remaining 97.3% showing no significant change. **(B)** Volcano plot displaying differentially expressed genes (DEGs) following IRF3 knockout, with red dots representing significantly upregulated genes (Z-score > 2, -log10(p) > 1.3). **(C)** Bar chart of the top 20 upregulated genes. **(D)** Bubble plot of GO enrichment analysis for upregulated genes, showing significant enrichment in immune-related biological processes (BP), cellular components (CC), and molecular functions (MF), such as regulation of T-cell activation, leukocyte cell-cell adhesion, and antigen receptor-mediated signaling. **(E)** Bubble plot of KEGG pathway enrichment analysis, demonstrating that upregulated genes are involved in immune signaling pathways and cell cycle/proliferation pathways.

To dissect the role of IRF3 in tumor cells, we first purified LUAD tumor cells from the epithelial cell population by assessing the expression of lung adenocarcinoma-specific markers, including NKX2-1, NAPSA, and CEACAM5 (**Supplementary Figure 4**). The scTenifoldKnk package was then employed to perform in silico IRF3 knockout experiments. IRF3 knockout induced differential expression of 2.7% of genes (**Figure 5A**). Notably, all significant differentially expressed genes were upregulated (**Figure 5B**; **Supplementary Table 9**), with top upregulated genes including SAMSN1, TRBC2, and CRIP2 (**Figure 5C**), which are primarily associated with T-cell activation and immune signaling. Functional enrichment analysis revealed that upregulated genes were enriched in immune-related biological processes, including regulation of T-cell activation, leukocyte cell-cell adhesion, and antigen receptor-mediated signaling pathways (**Figure 5D**). Pathway enrichment analysis further demonstrated that these genes were involved in the T-cell receptor signaling pathway, PD-L1 expression and PD-1 checkpoint pathway in cancer, and natural killer cell-mediated cytotoxicity (**Figure 5E**). These findings suggest that IRF3 may constrain pro-inflammatory and anti-tumor immune signaling in tumor cells. Additionally, enrichment in cell cycle and DNA replication pathways suggests a potential effect of IRF3 depletion on tumor cell proliferation (**Figure 5E**). Collectively, these transcriptomic signatures support a potential role of IRF3 in suppressing immune-activating gene expression and modulating proliferative molecular programs in tumor cells.

### 3.8 IRF3 in T/NK cells: negative regulation of pro-inflammatory signaling

**Figure 6:**
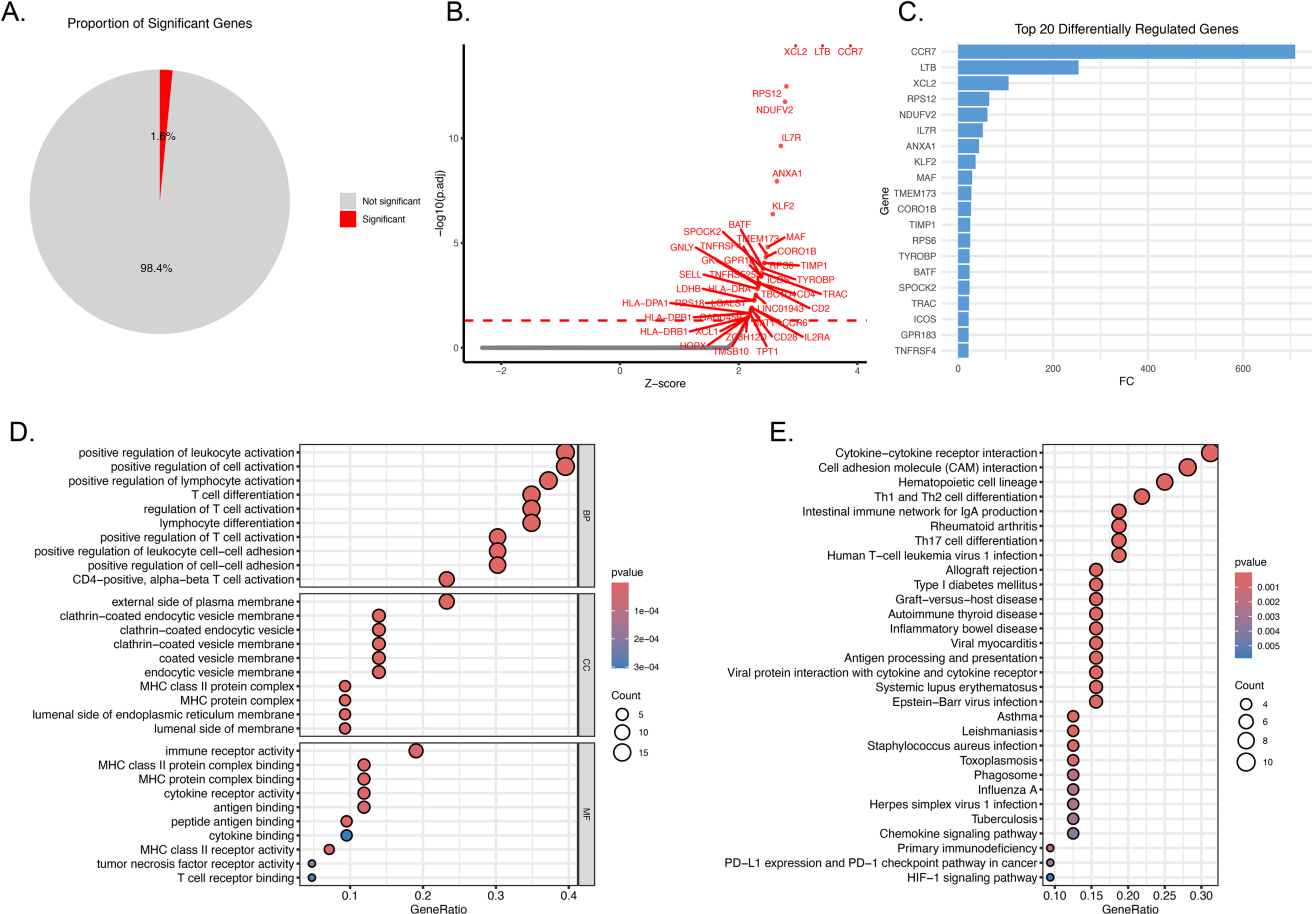
Virtual IRF3 knockout in T/NK cells triggers pro-inflammatory gene expression and immune pathway activation. **(A)** Pie chart showing that 1.6% of genes were differentially expressed following virtual IRF3 depletion, with the remaining 98.4% showing no significant change. **(B)** Volcano plot illustrating differentially expressed genes. **(C)** Bar chart of the top 20 differentially regulated genes. **(D)** GO enrichment analysis of upregulated genes, demonstrating significant enrichment in biological processes related to immune cell activation, including positive regulation of leukocyte activation, T-cell differentiation, and leukocyte cell-cell adhesion. The dot size represents gene count, color indicates p-value, and x-axis shows the GeneRatio. **(E)** KEGG pathway enrichment analysis, revealing activation of critical immune signaling pathways, such as cytokine-cytokine receptor interaction, cell adhesion molecule (CAM) interaction, antigen processing and presentation, and the PD-1/PD-L1 checkpoint pathway.

Virtual IRF3 knockout in T/NK cells induced differential expression of 1.6% of genes, with all significant differentially expressed genes also being upregulated (**Figures 6A, B**; **Supplementary Table 10**). The top 20 upregulated genes were dominated by immune mediators, including CCR7, LTB, RPS12 and NDUFV2 (**Figure 6C**). GO enrichment analysis revealed that upregulated genes were significantly enriched in biological processes linked to T-cell and leukocyte activation, including positive regulation of leukocyte activation, T-cell differentiation, and leukocyte cell-cell adhesion (**Figure 6D**). KEGG pathway analysis further demonstrated enrichment in critical immune signaling pathways, such as cytokine-cytokine receptor interaction, cell adhesion molecule (CAM) interaction, antigen processing and presentation, and the PD-1/PD-L1 checkpoint pathway (**Figure 6E**). These data demonstrate that IRF3 acts as a negative regulator of pro-inflammatory signaling in T/NK cells, with its depletion leading to the de-repression of immune-activating gene programs.

## 4 Discussion

In this study, we integrated multi-omics analyses and functional experiments to systematically investigate the prognostic value and immunoregulatory role of TRIM family genes in LUAD. We identified TRIM59 as an independent poor prognostic factor and uncovered a novel TRIM59-IRF3 regulatory axis that drives immune evasion and tumor progression. Our findings demonstrate that TRIM59 promotes IRF3 ubiquitination and proteasomal degradation, thereby inhibiting the STING-IRF3 pathway and shaping an immunosuppressive TME. Single-cell analyses further reveal context-dependent functions of IRF3 in tumor cells and tumor-infiltrating NK/T cells, highlighting the complexity of this regulatory axis.

The clinical significance of TRIM59 as an independent prognostic factor is consistent with its oncogenic role in multiple cancers (24–28). As an E3 ubiquitin ligase, TRIM59 exerts pro-tumor effects by regulating the stability of key proteins involved in diverse oncogenic processes, including ferroptosis, glycolysis, mitochondrial lipid metabolism, and the Notch signaling pathway. Consistent with these prior observations, our data demonstrate that TRIM59 is significantly upregulated in LUAD tumor tissues and its high expression correlates with shorter overall survival. This suggests that TRIM59 may serve as a novel prognostic biomarker for LUAD patients. Mechanismly, we found that TRIM59 promotes IRF3 ubiquitination and degradation, thereby inhibiting the STING-IRF3 pathway and shaping an immunosuppressive TME. Accumulating evidence has indicated the regulatory role of TRIM59 in innate immune system. A previous study reported by Kondo et al. (29) demonstrated that TRIM59 negatively modulates NF-κB and IRF3/7-mediated innate immune signaling under inflammatory and infectious conditions, which preliminarily established the inhibitory effect of TRIM59 on IRF3-related pathways. However, this work focused on physiological inflammation rather than malignant tumors, and the regulatory pattern between TRIM59 and IRF3 in lung cancer remains unclear. Herein, we identified TRIM59 as an E3 ligase that directly targets IRF3 for ubiquitin-dependent degradation in LUAD, extending the immune regulatory function of TRIM59 from inflammatory responses to cancer immunomodulation. Notably, TRIM59 has been reported to inhibit NSCLC cell autophagy and ferroptosis processes by promoting ubiquitination and degradation of TMEM164 (30), our study expands this by demonstrating its role in immune suppression. Collectively, our study uncovers a novel mechanistic layer underlying the oncogenic activity of TRIM59, highlighting its role as a key negative regulator of anti-tumor immunity in LUAD.

The cGAS–STING–IRF3 signaling cascade plays a central role in orchestrating antitumor immune responses (15, 16), with its activity tightly regulated by multiple posttranslational modifications, including acetylation, methylation, SUMOylation, and ubiquitylation (31, 32). While ubiquitylation-mediated regulation of cGAS and STING protein stability and functional activity has been well characterized in previous studies (33, 34), the regulatory role of ubiquitylation in IRF3 function during tumorigenesis remains poorly defined. However, numerous studies have elucidated the mechanisms underlying IRF3 ubiquitylation in antiviral immunity, which can provide theoretical frameworks for investigating IRF3 regulation in tumors. For instance, Dai et al. demonstrated that AXIN1 enhances antiviral responses by stabilizing IRF3 protein (35), and another study reported that DYRK4 promotes virus-triggered IRF3 activation and type I IFN induction via increasing IRF3 linear ubiquitylation and maintaining its stability (36). Notably, Yi et al. (37) validated the role of IRF3 in mediating innate immune activation and tumor-suppressive effects in NSCLC, highlighting the protective function of IRF3 against lung tumor progression. Nevertheless, the upstream post-translational mechanisms driving IRF3 downregulation have not been fully elucidated. In this study, we extend these findings to the tumor context by identifying TRIM59 as a novel E3 ubiquitin ligase that directly participates in regulating IRF3 ubiquitylation in LUAD. Our findings strongly support TRIM59 as a novel therapeutic target for modulating cGAS-STING-IRF3 pathway-mediated antitumor immunity.

Single-cell analyses provide unprecedented insights into the cell-type-specific functions of IRF3, which may be linked to the complex regulatory role of TRIM59 in shaping the TME of LUAD. In LUAD tumor cells, IRF3 is implicated in the modulation of both proliferative and immune-related genes. Transcriptomic evidence indicates that IRF3 depletion correlates with enhanced proliferative signatures and elevated immune-activating gene expression, implying a potential role in restricting tumor cell growth and limiting anti-tumor immune signaling. In contrast, IRF3 acts as a potential immune suppressor in T/NK cells. Following virtual IRF3 knockout, genes associated with immune activation, antigen presentation, and tumor homing were markedly upregulated, suggesting that IRF3 may intrinsically constrain immune effector programs. This context-dependent functionality of IRF3 highlights its opposing roles across distinct cellular compartments within the TME. Collectively, these observations highlight IRF3 as a central node orchestrating crosstalk between tumor cells and immune cells in LUAD, and its cell-type-specific functions provide a compelling rationale for targeted strategies that modulate the TRIM59-IRF3 axis. Such approaches hold the potential to simultaneously inhibit tumor proliferation and reinstate T/NK cell-mediated anti-tumor immunity, thereby remodeling the TME toward an immune-permissive state conducive to effective anti-tumor responses.

Limitations of our study include the relatively small sample size of scRNA-seq specimens and the lack of *in vivo* validation. Additionally, the exact ubiquitination sites on IRF3 targeted by TRIM59 remain to be identified. Notably, this study primarily focuses on LUAD, and we have not yet verified whether the TRIM59-IRF3 regulatory mechanism is conserved in other NSCLC subtypes, such as lung squamous cell carcinoma and large cell carcinoma. Future studies will focus on validating the TRIM59-IRF3 axis in larger clinical cohorts and preclinical models, as well as developing specific TRIM59 inhibitors. Furthermore, exploring the role of TRIM59 in other immune cell types and its potential crosstalk with other signaling pathways will provide a more comprehensive understanding of its role in LUAD progression.

## 5 Conclusion

In summary, our study identifies a novel cell-type-specific TRIM59-IRF3 regulatory axis that drives immune evasion and tumor progression in LUAD. TRIM59, an independent poor prognostic factor, promotes IRF3 ubiquitination and proteasomal degradation, inhibiting the IRF3-STING pathway and shaping an immunosuppressive TME. IRF3 exhibits context-dependent functions in tumor cells and T/NK cells, with TRIM59-mediated degradation disrupting this balance to favor tumor progression. These findings establish TRIM59 as a promising prognostic biomarker and the TRIM59-IRF3 axis as a therapeutic target for enhancing immunotherapy efficacy in LUAD.

## Data Availability

The original contributions presented in the study are included in the article/**Supplementary Material**. Further inquiries can be directed to the corresponding author.
