# Supplementary figures and images for: TRIM59 promotes immune evasion and tumor progression in lung adenocarcinoma via ubiquitin- proteasomal degradation of IRF3
Source: Front Immunol. 2026 May 20;17:1813280. doi: 10.3389/fimmu.2026.1813280 (PMC13230104; doi:10.3389/fimmu.2026.1813280)

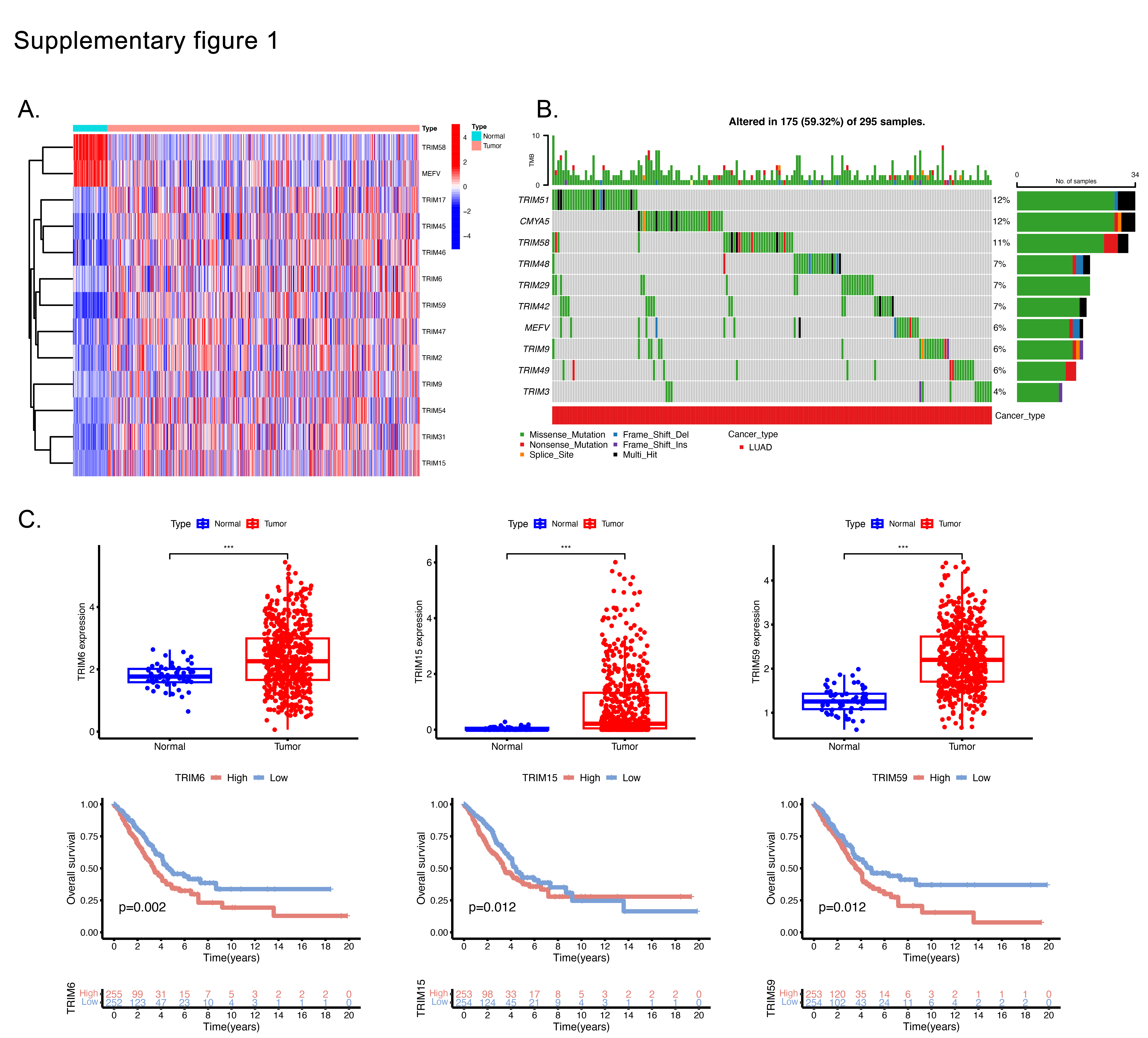

Supplement: Supplementary file 1 [file Image1.jpeg]

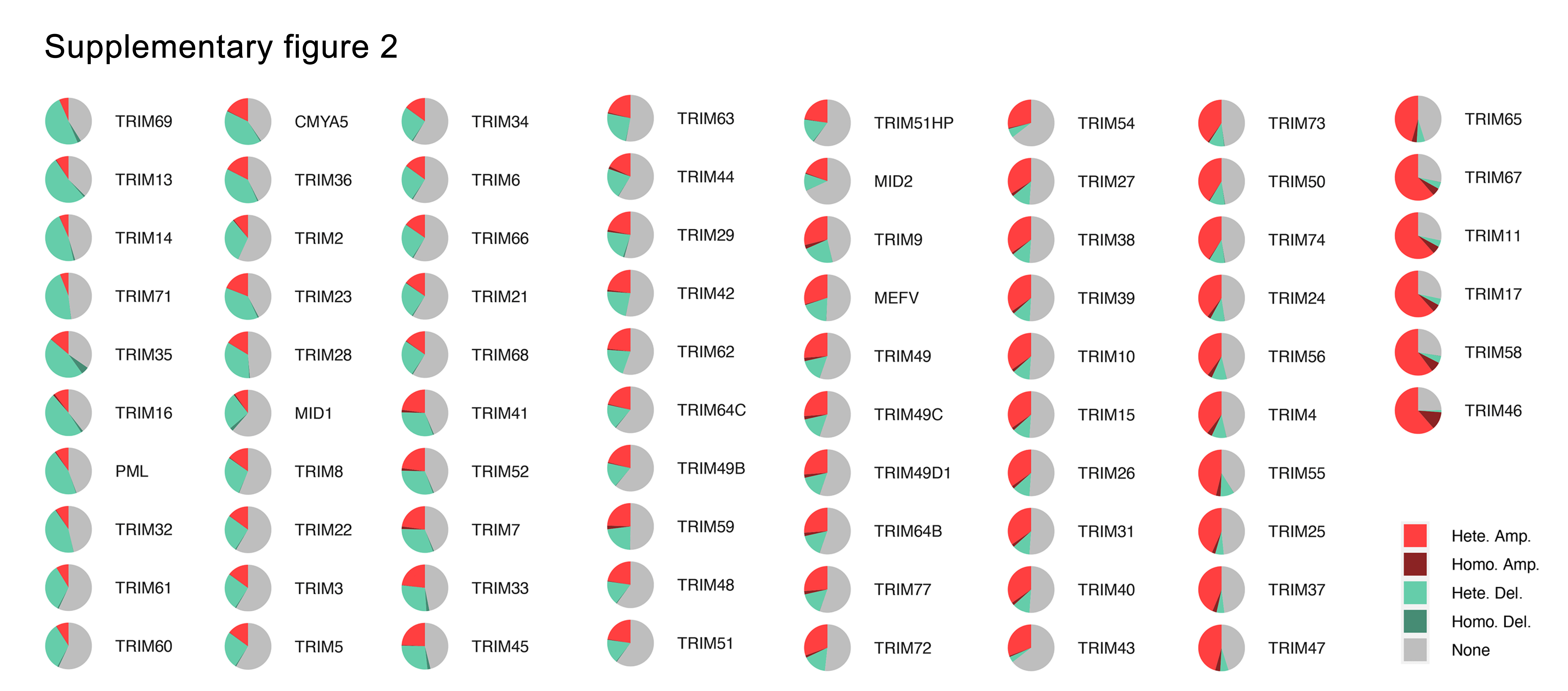

Supplement: Supplementary file 2 [file Image2.jpeg]

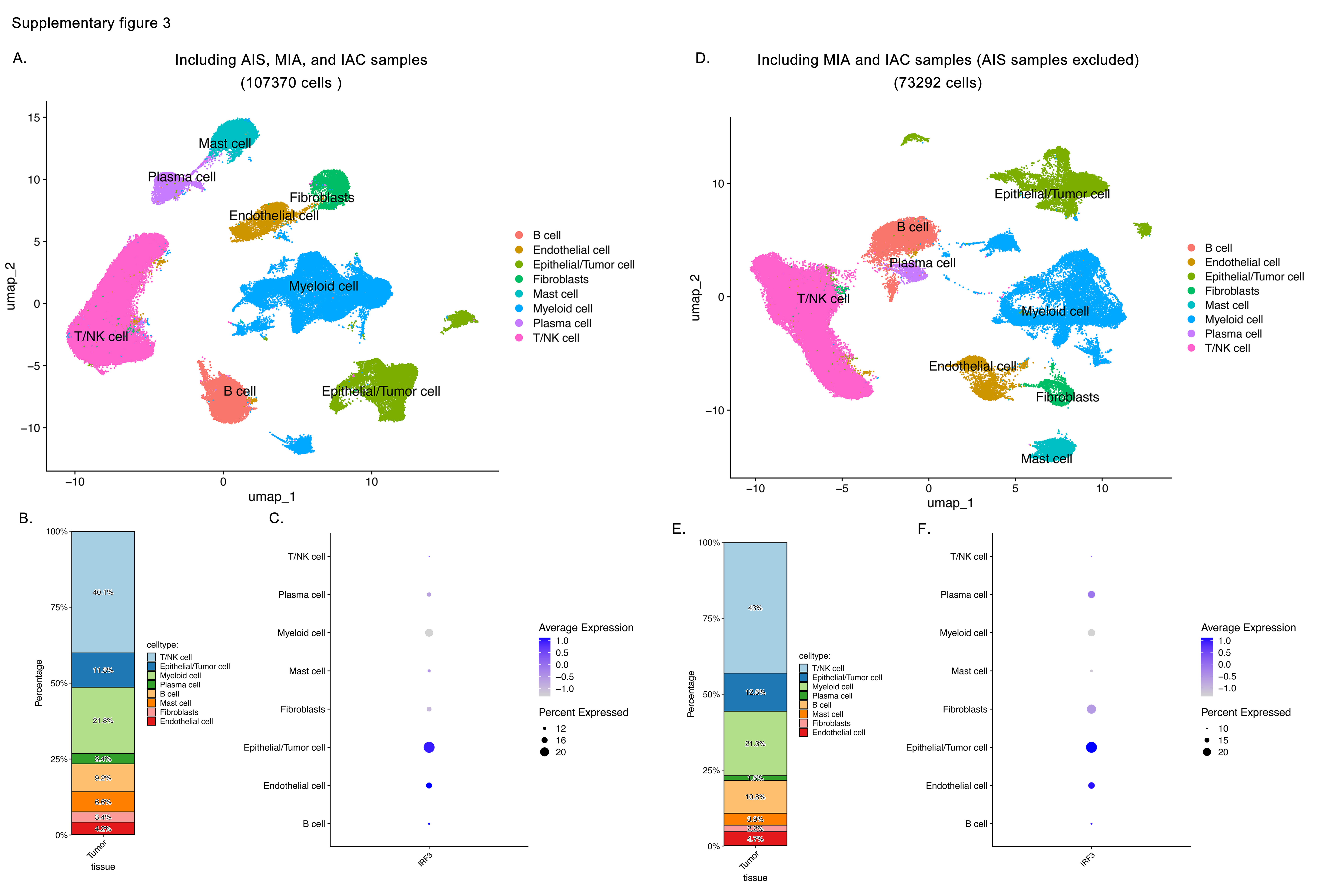

Supplement: Supplementary file 3 [file Image3.jpeg]

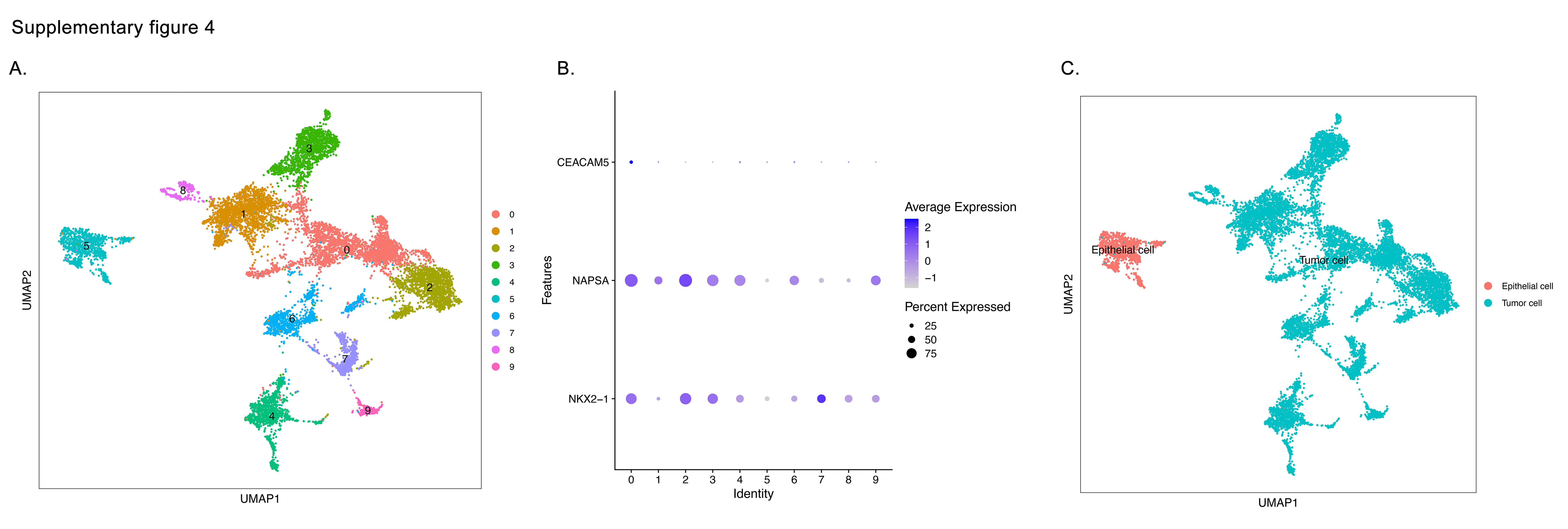

Supplement: Supplementary file 4 [file Image4.jpeg]
